# Hydrodynamic loading in concomitance with exogenous cytokine stimulation modulates differentiation of bovine mesenchymal stem cells towards osteochondral lineages

**DOI:** 10.1186/s12896-016-0240-6

**Published:** 2016-02-01

**Authors:** Stephen M. Goldman, Gilda A. Barabino

**Affiliations:** Interdisciplinary Bioengineering Graduate Program, Georgia Institute of Technology, Atlanta, GA, 30332 USA; G.W. Woodruff School of Mechanical Engineering, Georgia Institute of Technology, Atlanta, GA, 30332 USA; Department of Biomedical Engineering, City College of New York, 160 Convent Avenue, New York, NY, 10031 USA

**Keywords:** Mesenchymal stem cells, Fluid shear stress, Chondrogenesis, Osteogenesis, Bioprocessing, Biomanufacturing

## Abstract

**Background:**

Mesenchymal stem cells (MSCs) are viewed as a having significant potential for tissue engineering and regenerative medicine therapies. Clinical implementation of MSCs, however, demands that their preparation be stable and reproducible. Given that environmental and bioprocessing parameters such as substrate stiffness, seeding densities, culture medium composition, and mechanical loading can result in undirected differentiation of the MSC population, the objective of this study was to systematically investigate how hydrodynamic loading influences the differentiation of bone marrow-derived mesenchymal stem cells (MSCs) towards the osteochondral lineages both in the presence and absence of exogenous, inductive factors.

**Methods:**

Expanded bovine MSCs were suspended in 2.5 % agarose, cast in a custom mold, and placed into either static or one of two dynamic culture environments consisting of “high” and “low” magnitude shear conditions. Constructs were supplemented with varying concentrations (0, 1, 10, 100 ng/mL) of either TGF-*β*3 or BMP-2 throughout cultivation with tissue samples being collected following each week of culture.

**Results:**

In the absence of exogenous supplementation, hydrodynamic loading had little effect on cell phenotype at either magnitude of stimulation. When cultures were supplemented with BMP-2 and TGF-*β*3, MSCs gene expression progressed towards the osteogenic and chondrogenic pathways, respectively. This progression was enhanced by the presence of hydrodynamic loading, particularly under high shear conditions, but may point the chondrogenic cultures down a hypertrophic path toward osteogenesis reminiscent of endochondral ossification if TGF-*β*3 supplementation is insufficient.

**Conclusions:**

Moving forward, these results suggest bioprocessing conditions which minimize exposure of chondrogenic cultures to fluid shear stress to avoid undesirable differentiation of the MSC population.

**Electronic supplementary material:**

The online version of this article (doi:10.1186/s12896-016-0240-6) contains supplementary material, which is available to authorized users.

## Background

Due to their limited supply and decreased proliferative capacity, the sole use of autologous chondrocytes and osteoblasts for regenerative medicines is likely unsustainable [1]. Subsequently, mesenchymal stem cells (MSCs) have emerged as a clinically relevant cell source for regenerative medicine, due to their ease of procurement, multipotentiality, high proliferation rate, and ability to be expanded in vitro while maintaining a stable phenotype [2–4]. Directed differentiation of MSCs along various mesenchymal pathways can be achieved by manipulation of the cell culture environment including supplementation of culture medium with soluble morphogens [5–9], modulation of culture substrate stiffness [10], and external forces [11, 12]. Of particular interest are environmental approaches which might increase differentiation efficiency while reducing upstream bioprocessing costs for the purpose of large scale commercial operations.

A predominant challenge of the scaling operations required to process large numbers of cells and/or critically sized tissue constructs is the control of nutrient and waste transport from the cells/tissues during culture. To overcome these issues, a number of bioreactor concepts have been developed to provide the flow of culture media through [13], across [14], and around the constructs [15, 16]. As a result of the medium exchange, the constructs are concurrently nourished and exposed to hydrodynamic loading. Shear stress is known to cause varied effects on cell populations, including transmembrane ion leakage, as well as physiological and metabolic changes [17]. The presence of fluid shear stress, therefore, is an important environmental factor which may play an important role in the stability or instability of the MSC phenotype in culture. Furthermore, if the magnitude and spatiotemporal presentation of hydrodynamic loading can be controlled, it may represent a novel approach to modulating the efficiency of directed MSC differentiation. The primary objective of this study, therefore, was to determine the effect of uniform shear stress magnitude and duration on MSC gene expression through a panel of key differentiation markers along the osteochondral differentiation pathway. These genes were selected for their importance in orthopedic tissue engineering applications and potential to provide a window into the chondrogenic and osteogenic differentiation processes.

Given the well-documented sensitivity of mature chondrocytes and osteoblasts to the TGF-β superfamily [8], a secondary objective of this study was to examine the response of MSCs to varying magnitudes of superficial hydrodynamic shear stress in cultures supplemented with varying concentrations of TGF-β3 and BMP-2. Drawing on evidence that both bone and cartilage are mechanosensitive [18, 19] and mechanical stimuli are anabolic [20, 21], we hypothesized that hydrodynamic loading would increase the efficiency of MSC differentiation down the desired pathways as revealed through systematic changes in phenotypic markers. The scope of the study was limited to a range of hydrodynamic conditions within the reported interstitial flow regime of bone [22] and cartilage [23] with a view to determining an optimum for lineage specific differentiation. Additionally, the cytokine concentrations were varied by one order of magnitude in either direction from the most ubiquitous supplementation protocols found in the literature concurrently, to determine how hydrodynamic culture might minimize their necessity.

## Methods

### Materials

Unless specified otherwise, supplies and reagents were purchased from VWR International (West Chester, PA), Sigma (St. Louis, MO) or Invitrogen (Carlsbad, CA). Antibodies were from AbD Serotec (Raleigh, NC) or Abcam (Cambridge, MA).

### Experimental design

To elucidate the role of hydrodynamic loading in MSC differentiation towards the osteochondral lineages, we selected three magnitudes of fluid shear stress (0, 1, 10 dyn/cm^2^) to be applied in the presence of four levels of exogenous stimulation (0, 1, 10, 100 ng/mL) for two different cytokines (BMP-2, TGF-β3) resulting in 12 experimental groups which received some level of both hydrodynamic and exogenous stimuli, three groups which received only TGF-β3 stimulation of varying degrees, three groups which received only BMP-2 stimulation of varying degrees, and three unsupplemented groups which received only hydrodynamic stimulation of varying degrees (Additional file 1: Table S1). Samples from each experimental group were collected on a weekly basis for 2 weeks. Additional samples for each group were generated at the start of tissue culture (Week 0), but never subjected to any of the stated experimental conditions in order to generate a baseline for downstream analysis.

### Tissue culture

**Fig. 1:**
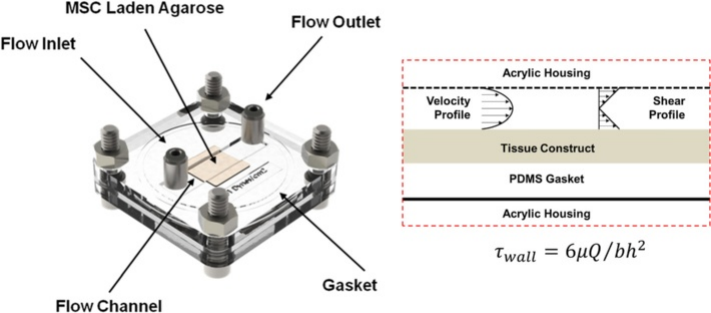
Tissue constructs were cultivated utilizing a custom built laminar flow chamber (*left*). Two separate devices with varying channel heights were produced such that parallel cultures of different hydrodynamic loading magnitudes could be simultaneously driven by a single peristaltic pump according to the relationship depicted (*right*)

MSCs isolated and characterized from the limbs three different animals post-mortem (see Additional file 2 Supplemental Methods, Additional file 3: Figure S1, Additional file 4: Figure S2) were mixed with sterile agarose solution such that the final concentration of MSCs in 2.5 % w/w agarose solution was 25 million cells/mL. Constructs were then cast into a polydimethylsiloxane [PDMS] mold and cultured either statically (0 dyn/cm^2^) or loaded in a custom flow chamber (Fig. 1) for dynamic culture at one of two wall shear stress (WSS) conditions: low shear (1 dyn/cm^2^) or high shear (10 dyn/cm^2^). To achieve the variation in shear stress at the wall (*τ*_*wall*_), the channel height (h) was varied between two different chamber designs while the kinematic viscosity (*μ*), Volumetric Flow Rate, and the channel width (b) were held constant. This approach allowed multiple flow loops from different experimental groups to be driven simultaneously by a single, multi-channel peristaltic pump (Masterflex, Cole Parmer, Vernon Hills, IL). Unsupplemented culture groups received a serum free basal media (high glucose DMEM, 1× PSF, 0.1 μM dexamethasone, 50 μg/mL ascorbate 2-phosphate, 40 μg/mL l-proline, 100 μg/mL sodium pyruvate, 1× insulin–transferrin–selenium [ITS]). All other experimental groups received the serum free basal media supplemented with a prescribed concentration (1, 10, or 100 ng/mL) or either TGF-β3 or BMP-2.

### mRNA expression

Real-time polymerase chain reaction (qRT-PCR) was used to quantify region specific gene expression within the constructs. Constructs were fixed in TRIzol, and RNA was isolated from the homogenized cell lysate through a series of rinse, elution, and centrifugation processes. The RNA samples were then reverse transcribed into cDNA using a QuantiTech Rev Transcription kit (Qiagen, Hilden, German) according to the manufacturer’s protocol. Gene expression for target mesenchymal lineage markers using custom-designed primers (Additional file 1: Table S2) with quantitative PCR amplification performed on a StepOnePlus™ Real-Time PCR System (Applied Biosystems) in the presence of SYBR Green/ROX master mix (Applied Biosystems). To determine fold regulation over Day 0 controls, the raw fluorescence data was processed using LinRegPCR (v12.11; http://www.hartfaalcentrum.nl) with glyceraldehyde-3-phosphate dehydrogenase (GAPDH) and β-Actin (ACTB) serving as the endogenous controls through geometric averaging [24]. Relative expression (*n* = 3 per condition and time point) of each target gene was calculated according to Eqs. 1–2, where *N*_0,*i*_ represents the initial concentration of the target gene, *N*_*q,i*_ represents the concentration of the target gene at the threshold, *E*_*i*_ represents the amplification efficiency of the polymerase chain reaction, and *C*_*q*_ is the selected threshold value.

1 $$ {N}_{0,i}={N}_{q,i}/{E}_i^{C_q} $$

2 $$ Fold\  Change={\left(\frac{N_{0,i}}{\sqrt{N_{0, GAPDH}{N}_{0, ACTB}}}\right)}_{sample}/{\left(\frac{N_{0,i}}{\sqrt{N_{0, GAPDH}{N}_{0, ACTB}}}\right)}_{control} $$

Endogenous controls were evaluated for each experimental group to ensure that their expression levels were not significantly altered across time or culture conditions.

### Histological analysis

For histological analysis, constructs were fixed in 10 % buffered formalin, embedded in paraffin and sectioned into 8 μm thick sections for the midsubstance of the construct. Sectioned samples were stained with Toluidine blue and Alizarin Red per established protocols. For immunofluorescence, sections were incubated with a citrate buffer heated to 99 °C for 30 min to retrieve antigens, and allowed to cool to room temperature. The samples were then incubated in blocking buffer for 30 min and primary rabbit anti-bovine antibodies (1:100, Abcam, Cambridge, MA) for Collagen types I, II, and X at 4 °C overnight. Sections were then washed three times in PBS and with DyLight®594 goat anti-rabbit secondary antibodies (1:200, Abcam, Cambridge, MA) for one hour at room temperature. Finally, samples were washed and mounted with Vectashield with DAPI and visualized on a Nikon Ti Eclipse inverted fluorescence microscope (Nikon Instruments, Inc., Melville, NY), with representative images captured using a CoolSNAP HQ2 CCD camera (Photometrics, Tucson, AZ).

### Statistical analysis

Independent experiments produced construct samples for RT-qPCR and immunohistochemistry (*N* = 3 per group). Gene expression is presented as the mean fold change ± SEM with statistically significant differences defined as *p* <0.05 using two-way ANOVA with Bonferroni post-hoc tests for multiple comparisons.

## Results

### Stability of gene expression in unsupplemented cultures

**Fig. 2:**
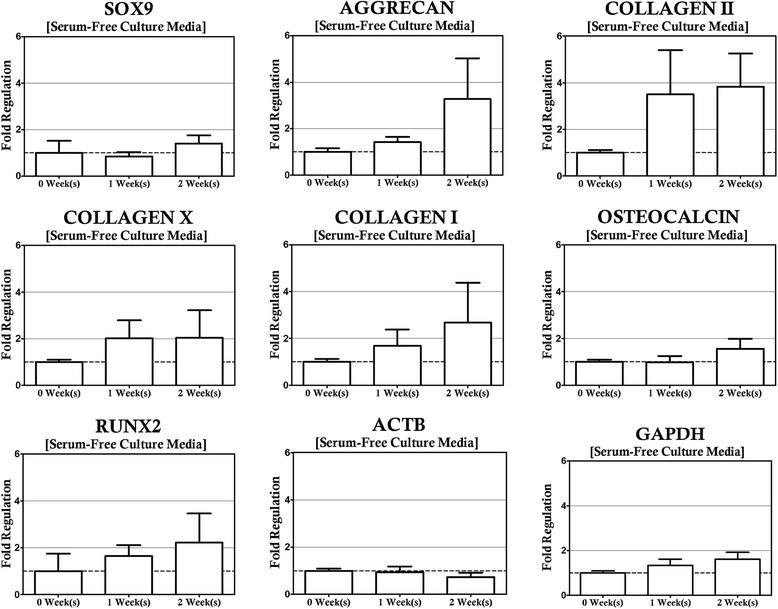
Gene Expression profiles were determined for unsupplemented, static cultures via RT-qPCR. No statistically significant regulation of the genes in the panel was observed

In order to control for the effect of hydrodynamic loading on the gene expression profile of MSCs, a round of control experiments was performed to assess the stability of the MSC transcriptome in unsupplemented, static, three dimensional culture over the course of 2 weeks of cultivation (Fig. 2). None of the genes measured exhibited significant regulation over the time course of the experiment with respect to the initial expression profile, indicating three dimensional culture in isolation of other factors was not a significant contributor to differentiation of the MSCs toward the desired lineages and that this culture format represents a suitable control for differentiation studies. This was an important realization, as there is evidence in the literature that subtle changes in culture conditions, such as the transition from monolayer to three-dimensional culture represented here can induce phenotypic changes in stem cell populations [10, 11, 25], particularly as a significant contributor to chondrogenesis [26]. It is important to note, however, that many of the protocols from the prior art depend on pellet culture whereas this study is dependent on the encapsulation of the MSCs in a three dimensional agarose hydrogel. The introduction of the hydrogel material provides additional barriers to communication by cell to cell contact, a factor known to play a role in chondrogenesis [27], and the deviation of the observations produced between these different systems may exist due to the relative differences in cell density between the two culture types. It is also noteworthy that the seeding density of the constructs was not varied in this study. It is possible seeding density may also play a role in this observation, as prior literature indicates seeding density can have an impact on ECM deposition in MSC-based tissue constructs [28, 29]. While regulation of the chondrogenic markers herein was not found to be significant these factors may play a role in the weak upward trend of COL2A1 with time in culture. Nevertheless, this effect was small and our observations confirmed the utility of this culture condition as a suitable control for our subsequent hydrodynamic culture studies aiming to determine the effect of exogenous cytokine supplementation on the mRNA expression profiles of this MSC population.

### Effect of hydrodynamic loading on unsupplemented cultures

**Fig. 3:**
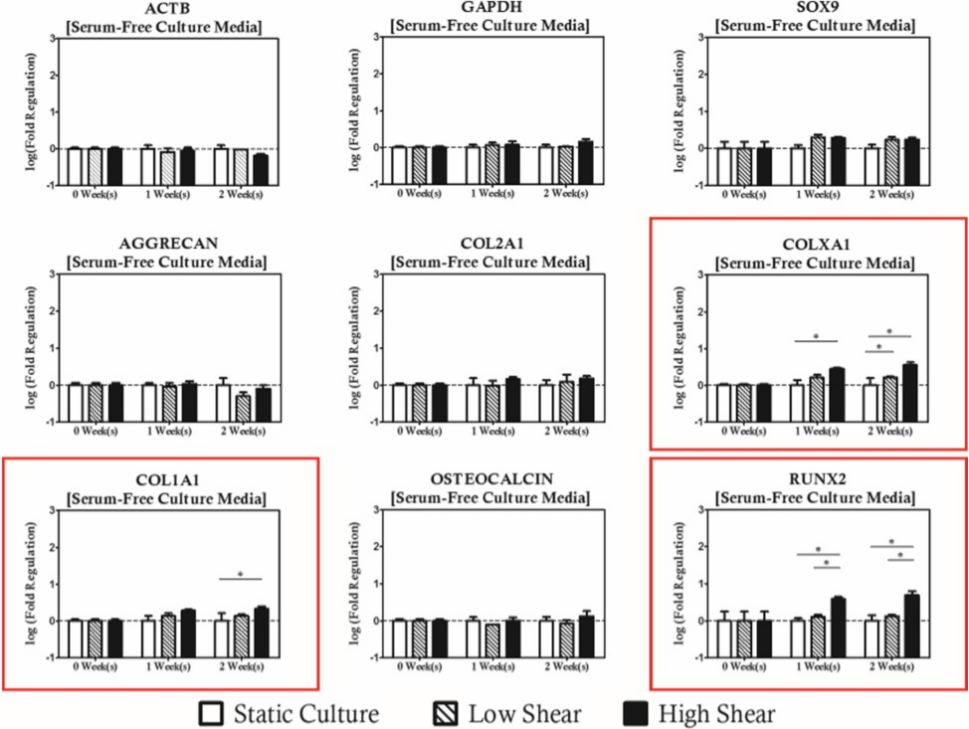
Hydrodynamic loading induced changes in gene expression of several osteochondral markers even in the absence of exogenous cytokine supplementation. Genes with statistically significant regulation are highlighted with a *red box*. Statistically significance is indicated by *asterisks*

With a suitable control group established, the first step in addressing the potential of hydrodynamic loading as a differentiation tool was to culture MSC based tissue constructs under laminar flow profiles with nominal shear stress magnitudes of 1 and 10 dyn/cm^2^ and to compare the expression of a panel of genes spanning the phenotypic diversity of cells along the endochondral ossification pathway to the previously discussed static controls. When hydrodynamic culture was introduced as a variable to the three-dimensional, serum-free cultures (Fig. 3), no significant regulation of the chondrogenic gene panel was observed, significant upregulation of the osteogenic transcription factor (*RUNX2*), and two of the three collagens investigated (*COL1Α1* and *COLXΑ1*) occurred under high shear conditions. *RUNX2* and *COLXA1* were both upregulated early in cultivation (1-week) and remained elevated relative to both the time matched static controls and the low shear treatment. At the 2-week time point, upregulation of *COL1A1* was considered significant relative to the static cultures. Interestingly, no significant difference in gene expression was observed with lower magnitude hydrodynamic loading, indicating that magnitude of shear in the absence of exogenous cytokine supplementation is not inconsequential. While these changes are considered statistically significant, the nominal change in expression of these genes was of less than one order of magnitude from the expression profile measured in the cell source population. When compared to the magnitude of impact of cytokine supplementation on gene expression when controlled for culture duration (>2 orders of magnitude difference), this effect is not likely to be useful as a tool for directed differentiation. At the same time, however, this finding suggests that great care should be taken to minimize the hydrodynamic loading applied to MSC expansion cultures in upstream bioprocessing procedures to prevent non-specific induction of undesirable phenotypes.

### Effect of cytokine supplementation on differentiation markers

While hydrodynamic loading in isolation of exogenous supplementation is not sufficiently potent to control differentiation in a selective manner, the results of our initial studies in unsupplemented, serum-free cultures suggested that hydrodynamic loading may be useful as when presented in concert with morphogens with a known inductive capacity. To investigate this possibility, we analyzed the expression profiles of statically cultured constructs which received either TGF-β3 or BMP-2 supplementation at a concentration of 1, 10 ng/mL, or 100 ng/mL for the purpose of inducing a chondrogenic or osteogenic phenotype, respectively. The resident MSCs tend towards expression of chondrogenic markers as function of time in culture and concentration of exogenous cytokine supplementation for both BMP-2 and TGF-β3 supplementation.

**Fig. 4:**
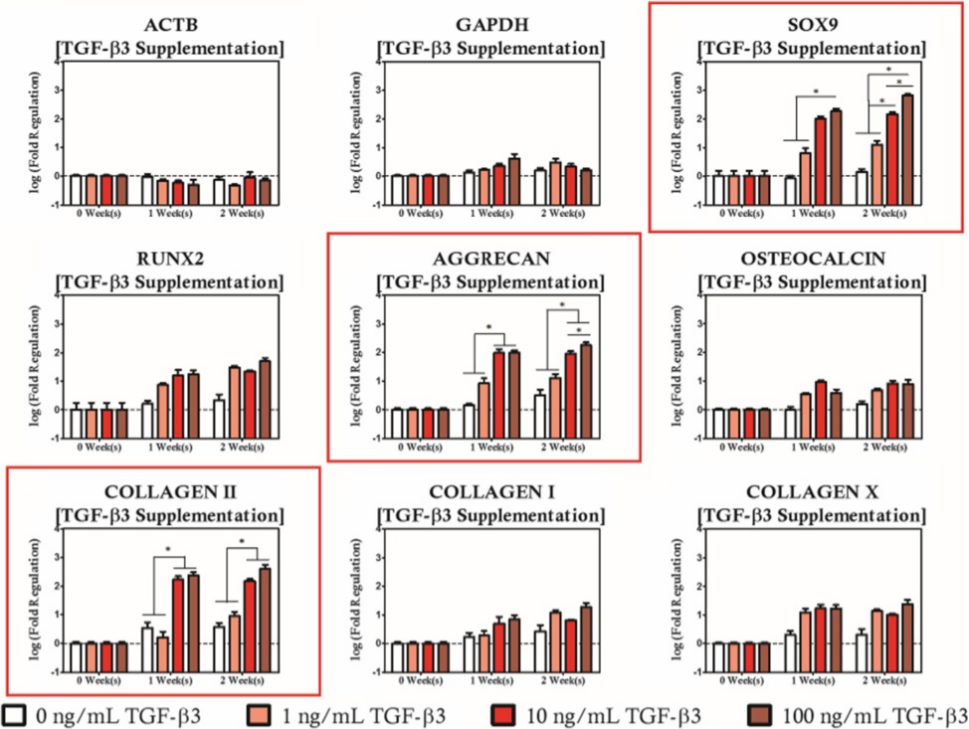
TGF-β3 supplementation modulates expression of chondrogenic markers without significantly altering the expression profile of the osteogenic panel. Genes with statistically significant regulation are highlighted with a *red box*. Statistically significance is indicated by *asterisks*

For TGF-β3 supplemented cultures, the expression of all three chondrogenic markers increased significantly relative to the unsupplemented control group for culture durations of at least 2 weeks provided the culture medium was supplemented with TGF-β3 at a concentration of at least 10 ng/mL while differences in expression of the chondrogenic markers for cultures supplemented at concentrations lower than 10 ng/mL were not considered significant (Fig. 4). After two weeks of culture, *SOX9* was expressed in a concentration dependent manner as the greatest change in expression relative to the source cell population occurred in the 100 ng/mL supplementation group (667 fold) which was significantly higher than the 10 ng/mL supplementation group (145 fold), which in turn was significantly greater than the 1 ng/mL supplementation group (12.7 fold). A similar trend in *AGGRECAN* and *COL2A1* expression was observed as increases in *AGGRECAN* expression relative to the source cell population was highest in the 100 ng/mL supplementation group (181 fold), which was statistically indeterminate from the 10 ng/mL supplementation protocol, but significantly higher than 1 ng/mL or lower concentration supplementation protocols. Likewise, *COL2A1* expression reached a maximum among the static cultures when TGF-β3 supplementation was provided at a concentration of 100 ng/mL (403 fold) for a period of 2 weeks. Considering the expression of undesirable hypertrophic and osteogenic genes, we found comparable mRNA levels among all TGF-β3 supplementation protocols. Additionally, the expression level of the hypertrophic and osteogenic markers are statistically indeterminate from the unsupplemented controls.

**Fig. 5:**
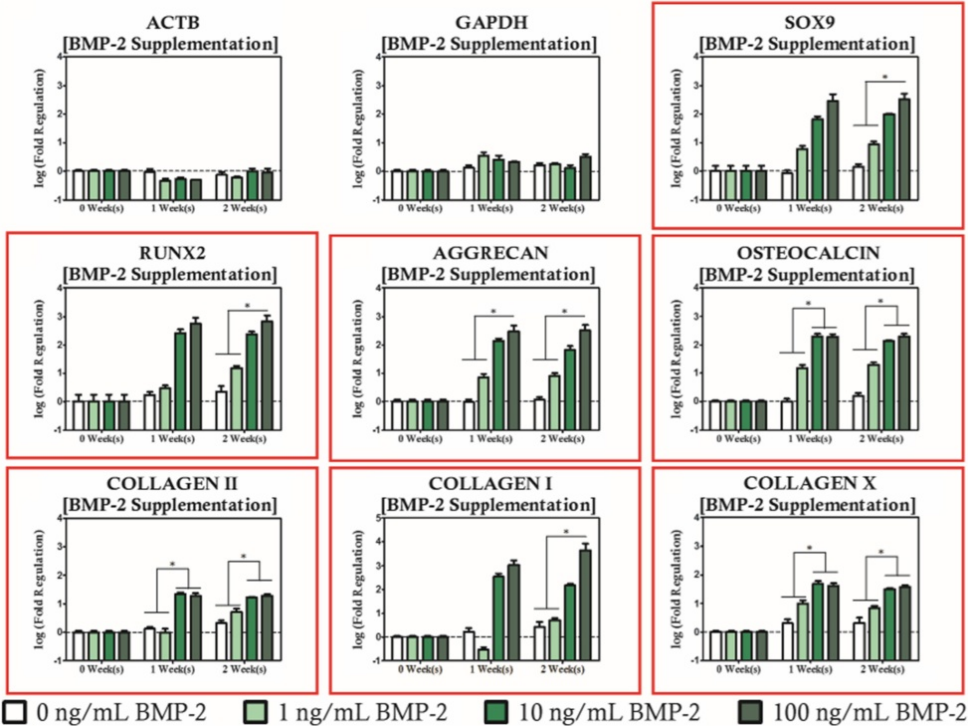
BMP-2 supplementation modulates expression of both chondrogenic and osteogenic markers. Genes with statistically significant regulation are highlighted with a *red box*. Statistically significance is indicated by *asterisks*

For BMP-2 supplemented cultures, upregulation of genes from both the chondrogenic and osteogenic panels at high cytokine concentrations was evident (Fig. 5). When the culture medium was supplemented with BMP-2 in concentrations in excess of 10 ng/mL, the entire chondrogenic gene panel (*SOX9*, *AGGRECAN*, and *COL2A1*) was upregulated relative to duration matched cultures receiving BMP-2 supplementation at a concentration of 1 ng/mL or less. Hypertrophic marker *COLXΑ1* also showed increases with respect to duration matched unsupplemented controls as BMP-2 concentration was increased. Regarding the osteogenic gene panel, *OSTEOCALCIN* was upregulated in cultures supplemented at 10 ng/mL or greater relative to duration matched cultures receiving 1 ng/mL or less for each culture period studied. *RUNX2* and *COL1A1* were also upregulated relative to the low supplementation groups (0 and 1 ng/mL), but only when BMP-2 supplementation was provided at a concentration of at least 100 ng/mL for a period of 2 weeks.

### Effect of hydrodynamic loading on cytokine supplemented cultures

Both BMP-2 and TGF-β3 produced strong differentiation of the MSC population utilizing the static culture platform, and provided a baseline for normalization of the hydrodynamically loaded cultures to control for the independent effect of the cytokines so that we might investigate whether stimulation via hydrodynamic loading can induce a synergistic effect on the gene expression profile of the differentiating cell population.

**Fig. 6:**
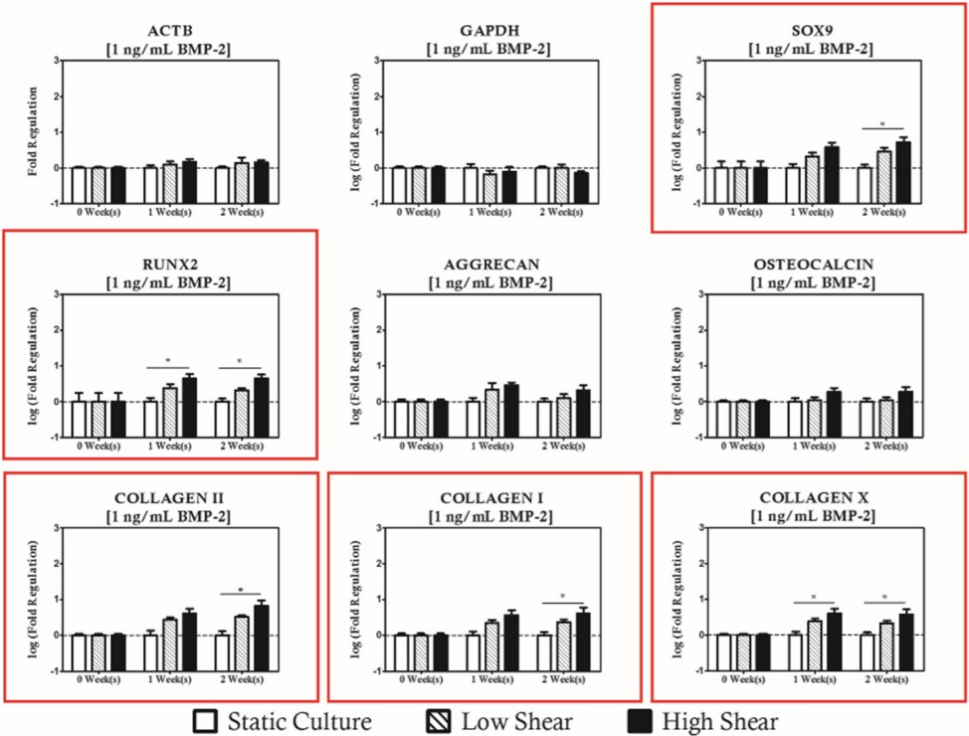
Even at low levels of BMP-2 supplementation, significant regulation of collagens and osteochondral transcription factors is observed. Genes with statistically significant regulation are highlighted with a *red box*. Statistically significance is indicated by *asterisks*

**Fig. 7:**
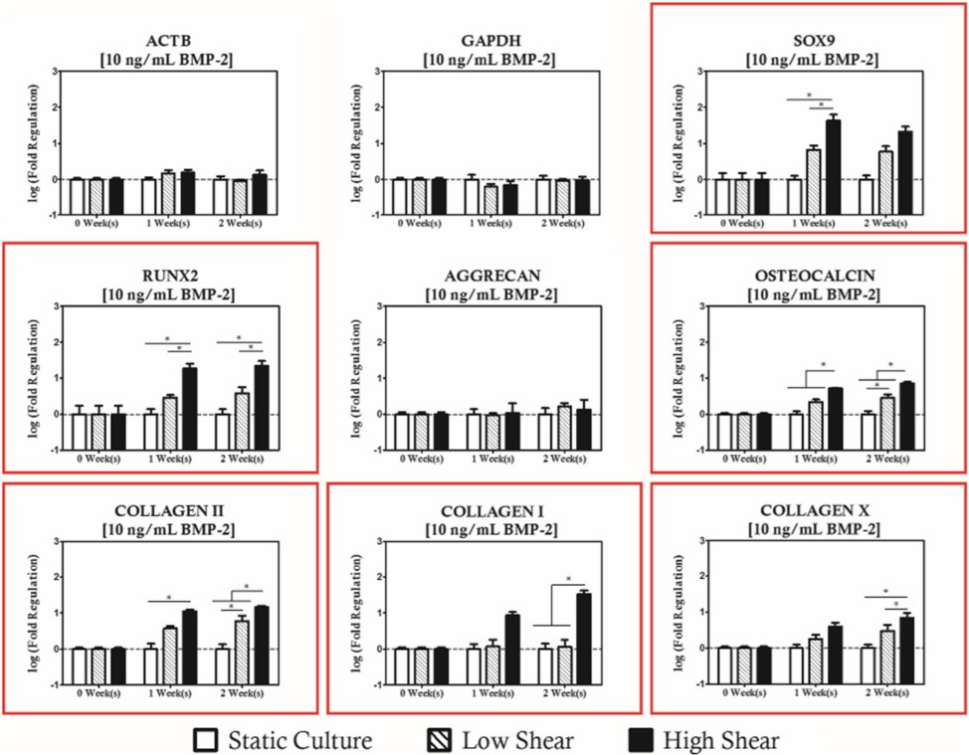
Transcription factors and collagen modulation is maintained in cultures supplemented with 10 ng/mL. Osteocalcin regulation is also observed under high shear conditions, suggesting a commitment to the osteogenic differentiation pathway. Genes with statistically significant regulation are highlighted with a *red box*. Statistically significance is indicated by *asterisks*

**Fig. 8:**
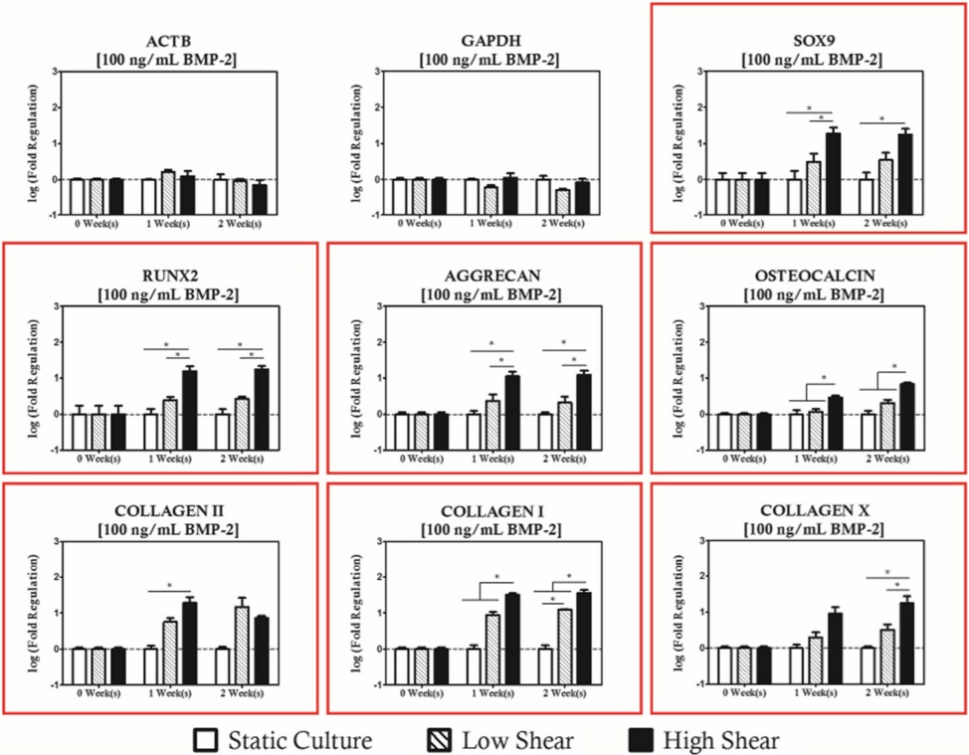
When BMP-2 is supplemented at a high level (100 ng/mL) shear stress is a significant modulator of all chondrogenic and osteogenic markers studied. Genes with statistically significant regulation are highlighted with a *red box*. Statistically significance is indicated by *asterisks*

BMP-2 supplemented cultures exhibited a strong shear coupling with respect to expression of *SOX9*, *RUNX2*, and all of the collagens studied, and was strongly biased towards high magnitude loading protocols (Figs. 6, 7 and 8). It is apparent that *RUNX2* was strongly upregulated for all BMP-2 supplementation protocols with concurrent high magnitude hydrodynamic loading as evidenced by significant increases relative to time-matched static controls at each time point investigated as well as significantly high expression relative to the low magnitude loading when BMP-2 concentration was at least 10 ng/mL. In addition to changes in expression of *RUNX2*, it was also observed that *COL1A1* was upregulated for the high shear condition groups. For the 1 ng/mL BMP-2 group, *COL1A1* expression was significantly higher in the high shear group compared to the static controls at two weeks of culture. When the concentration was raised to 10 ng/mL (Fig. 7) it was observed that the behavior was sustained in addition to being significantly higher than the low magnitude loading group. When BMP-2 supplementation was provided at a concentration of 10 ng/mL or lower, there was no significant difference in expression between the static and low magnitude hydrodynamic groups for either *RUNX2* or *COL1A1*. When BMP-2 supplementation was increased to 100 ng/mL (Fig. 8), however, it was observed that *COL1A1* was significantly upregulated in the low magnitude loading group relative to the static control after 2 weeks of culture. Additionally, hydrodynamic modulation of *OSTEOCALCIN* expression was observed for the first time in these studies in the high magnitude loading group relative to the static control after 2 weeks of culture in cultures receiving at least 10 ng/mL of BMP-2.

Regarding the chondrogenic markers, *SOX9* was upregulated in high magnitude loading culture relative to the static control for all BMP-2 supplementation groups, but interestingly this effect was only considered significant at the 1-week time point. *COL2A1* expression was observed to increase in high magnitude hydrodynamic cultures as well, as evidenced by significant increases relative to static controls at the 2-week time point for cultures receiving 1 ng/mL of BMP-2 and at both time points for culture receiving at least 10 ng/mL of BMP-2. This effect also appears to be sensitive the magnitude of hydrodynamic loading as significant differences were observed between the static cultures and low magnitude cultures as well as between the low and high magnitude cultures. Differences in *AGGRECAN* expression were only considered significant under high shear and high supplementation. It is also worth noting that expression of hypertrophic marker *COLXA1* was significantly increased in the high magnitude loading group after 2 weeks of culture for all BMP-2 supplementation protocols relative to the static control for the low concentrations (1 ng/mL) and to both low magnitude and static cultures at elevated concentrations of BMP-2 (10 and 100 ng/mL).

These results are not very surprising in light of the results from unsupplemented, hydrodynamically loaded construct group as two osteoinductive agents, hydrodynamic loading and BMP-2 supplementation, are at work simultaneously in these protocols. While the slight chondrogenic character of these cultures is not desirable, it is worth noting that modulation of chondrogenic markers (*SOX9*, COL2Α1) at high shear was of less than an order of magnitude and the order of the baseline control expression of these genes was considerably lower than their chondrogenic counterparts. *COLXΑ1* expression increased by an order of magnitude over culture period and supplementation matched static controls for both low and high shear conditions at two weeks when cultures were supplemented with 100 ng/mL of BMP-2. While the inductive impact of hydrodynamic loading is not as great in magnitude as that of BMP-2 supplementation at high levels (one order of magnitude change vs three orders of magnitude), it none the less is an important modulator of osteogenic induction as no significant difference was observed between static cultures supplemented at 100 ng/mL and cultures supplemented at 10 ng/mL that were also subjected to high magnitude hydrodynamic loading in terms of total gene expression relative to the initial MSC population.

**Fig. 9:**
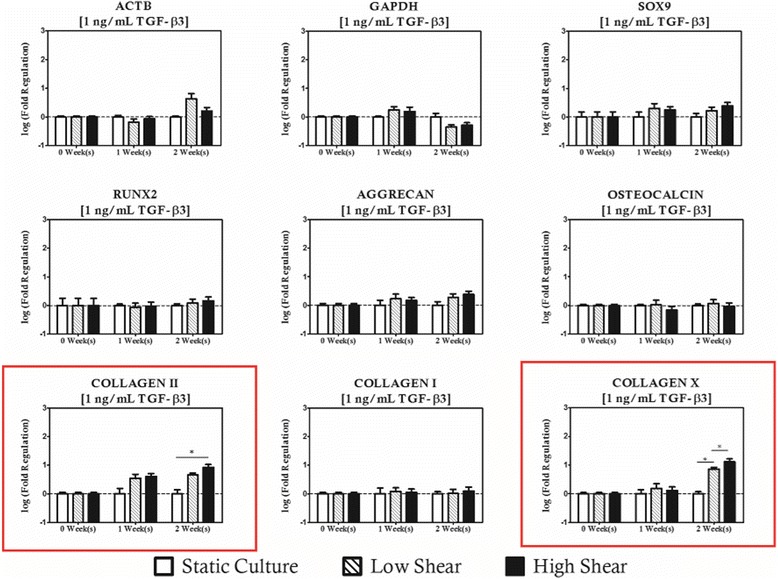
Hydrodynamic loading has limited impact on MSC based constructs with cultivated with low concentrations of TGF-β3 (1 ng/mL). Modulation of COL2A1 was considered significant with high magnitude hydrodynamic loading after 2 weeks of culture relative to time and concentration matched controls. Noticeably, the regulation of osteogenic genes with shear observed in unsupplemented controls disappears. Genes with statistically significant regulation are highlighted with a *red box*. Statistically significance is indicated by *asterisks*

**Fig. 10:**
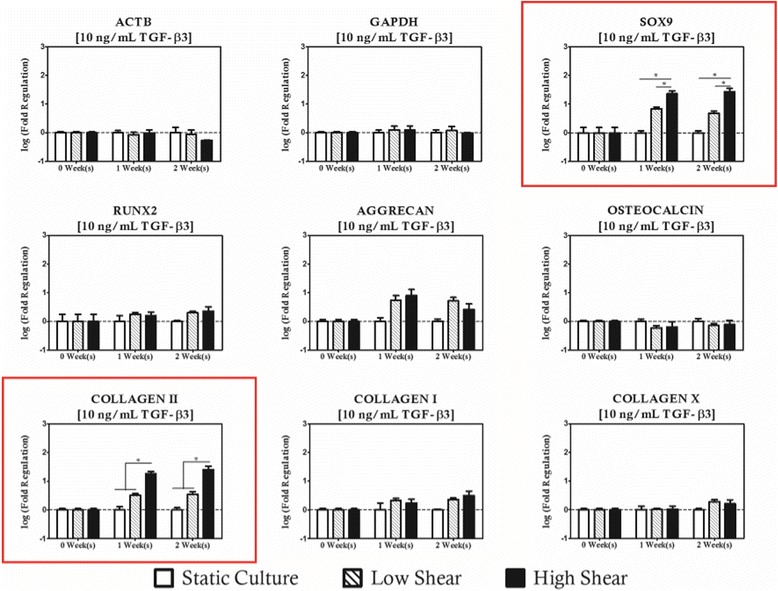
TGF-β3 supplementation of 10 ng/mL is the most ubiquitous supplementation protocol for chondrogenic cultures found in the literature. When hydrodynamic loading is introduced in concert at these levels of exogenous supplementation, SOX9 and COL2A1 are modulated in high shear environments. Osteogenic markers remain at levels comparable to static controls. Genes with statistically significant regulation are highlighted with a *red box*. Statistically significance is indicated by *asterisks*

**Fig. 11:**
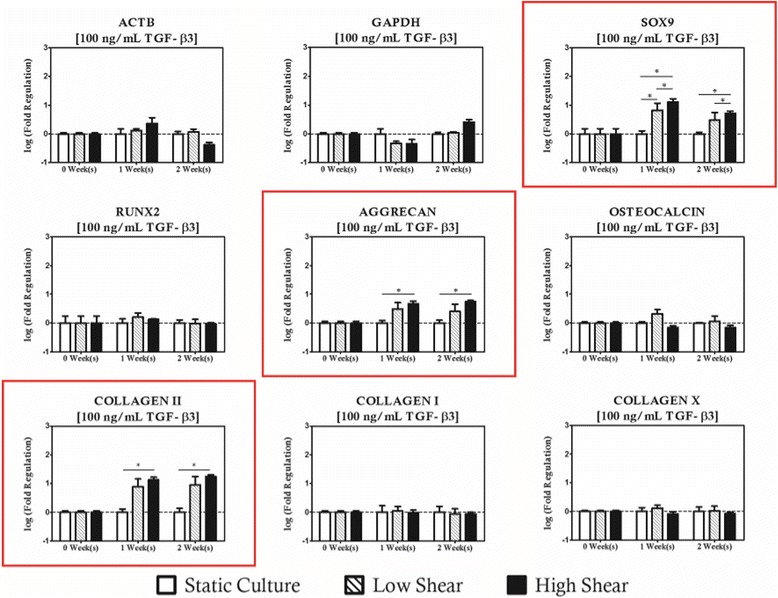
High levels of TGF-β3 supplementation results in strong upregulation of chondrogenic genes in the presence of hydrodynamic loading. Genes with statistically significant regulation are highlighted with a *red box*. Statistically significance is indicated by *asterisks*

When hydrodynamic stimulation was introduced in concert with TGF-β3 supplementation, it was observed that *COL2A1* was upregulated relative to duration and supplementation group matched controls when the hydrodynamic loading condition was high (10 dyn/cm^2^) and TGF-β3 concentrations were low indicating a mild synergistic effect on the chondrogenic induction of the resident cell population (Fig. 9). When the supplementation protocol was increased to 10 ng/mL, high shear cultures resulted in upregulation of both *SOX9* and *COL2A1*. No regulation of hypertrophic or osteogenic markers was observed at this supplementation level, and interestingly there was no effect on chondrogenic markers in low magnitude hydrodynamic cultures (Fig. 10). Upon increasing the TGF-β3 protocol to 100 ng/mL, shear magnitude dependent modulation of all three chondrogenic genes studied was observed. The chondrogenic panel was upregulated under high magnitude shear conditions relative to static controls after 1 week of culture and remained significantly high for subsequent culture durations (Fig. 11). There was no statistical difference between low and high shear conditions for *COL2A1* and *AGGRECAN* expression, but there was a shear magnitude dependency observed for *SOX9*.

Interestingly, changes in expression of the osteogenic gene panel were not considered significant for any of the hydrodynamic regimes studied. Expression of COLXΑ1, however, was upregulated relative to concentration matched static controls when conditions were such that high shear magnitudes (10 dyn/cm^2^) were paired with low (1 ng/mL) concentrations of TGF-β3 for a period of at least 2 weeks. No significant changes in COLXA1 were observed with moderate or high TGF-β3 supplementation. These observations hint at two potentially useful characteristics of this approach. First, chondrogenic differentiation is clearly positively influenced by the presence of hydrodynamic loading when presented in concert with at least 10 ng/mL of TGF-β3, and that TGF-β3 signaling appears to have an inhibitory effect the on the osteoinductive role of high magnitude hydrodynamic loading observed with the other supplementation protocols studied herein.

### Histology & immunofluorescence

**Fig. 12:**
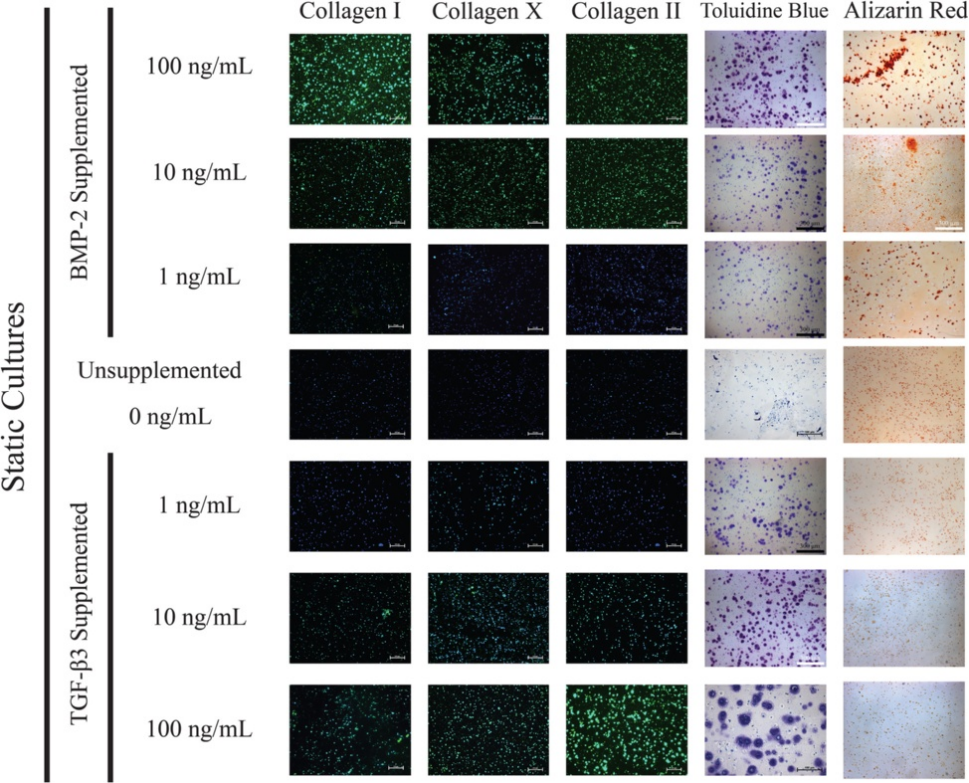
Histological and Immunofluorescence analyses of static cultures suggest increasing osteogenic character with BMP-2 supplementation and increasing chondrogenic character with TGF-β3 supplementation

**Fig. 13:**
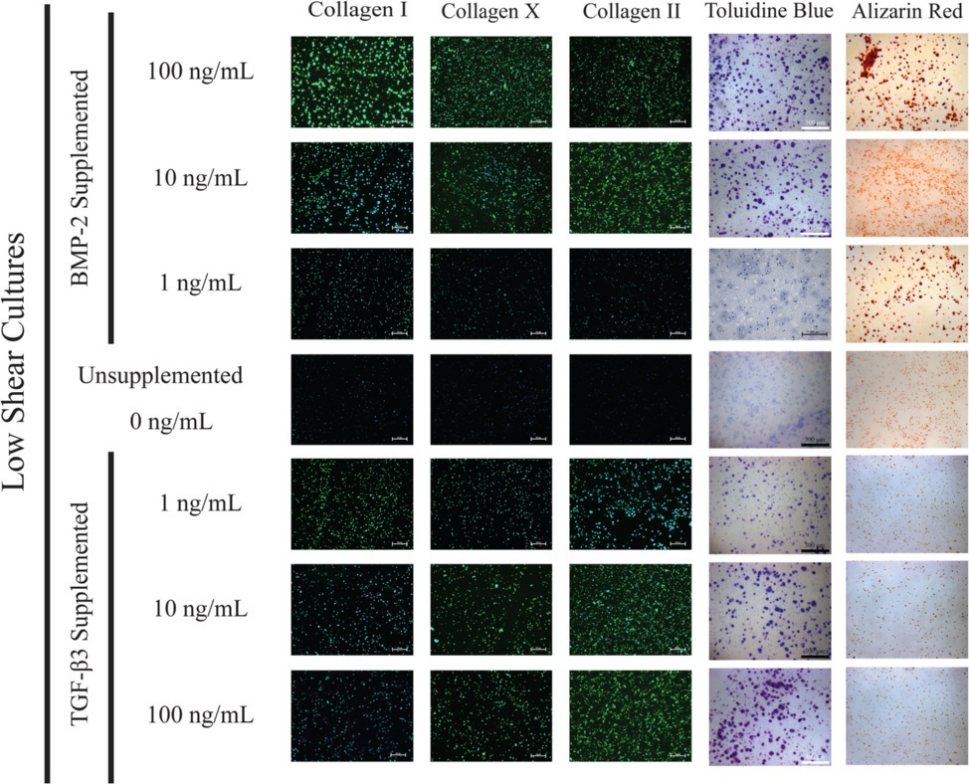
Histological and Immunofluorescence analyses of low shear cultures suggest increasing osteogenic character with BMP-2 supplementation and increasing chondrogenic character with TGF-β3 supplementation

**Fig. 14:**
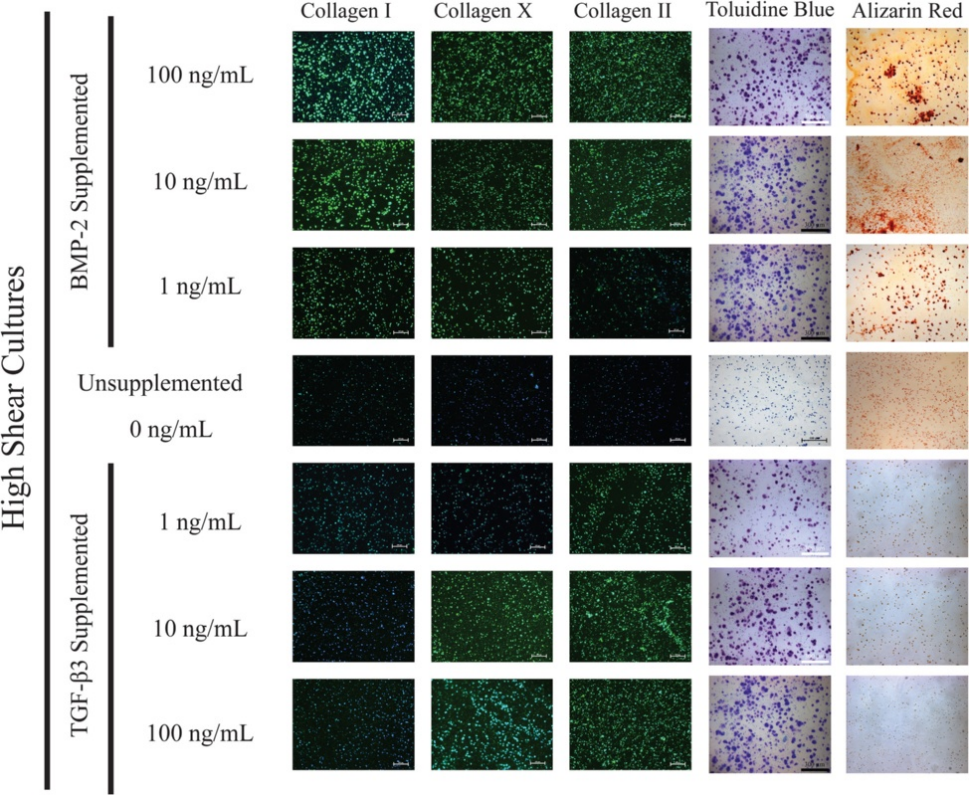
Histological and Immunofluorescence analyses of high shear cultures suggest increasing osteogenic character with BMP-2 supplementation and increasing chondrogenic character with TGF-β3 supplementation

Histological staining and immunofluorescence of 2-week culture samples qualitatively supports the gene expression profiles observed through PCR (Figs. 12, 13 and 14). Toluidine Blue staining indicates increasing expression of sulfated glycosaminoglycans with increases in both BMP-2 and TGF-β3 supplementation, while Alizarin Red staining shows greater staining with increased BMP-2 supplementation. Alizarin Red staining was relatively uniform for TGF-β3 supplemented cultures for all hydrodynamic and supplementation protocols tested. Immunofluorescence indicates increasing Collagen type I and Collagen type II expression in the BMP-2 and TGF-β3 supplemented cultures of increasing concentration, respectively. Trends in collagen expression between shear conditions are less clear, but there appears to be more total collagen in BMP-2 supplemented cultures on the whole, and total collagen expression appears to increase with hydrodynamic loading.

## Discussion

As the predominate source of cells for tissue engineered constructs has shifted from terminally differentiated primary cells towards progenitor cells of various differentiation potentials, the ability to spatiotemporally exert epigenetic control over the differentiation of stem cells within a tissue engineered construct has become desirable as a means to better reproduce native tissue complexity and reduce cultivation costs associated with traditional differentiation protocols. Subsequently, we decided to focus herein on the impact of the hydrodynamic environment on MSC differentiation due to its essential role in nutrient exchange during in vitro cultivation. The purpose of this study was to investigate how modulation of hydrodynamic loading affects the stability of the MSC phenotype during serum-free tissue culture and how this approach might enhance differentiation efficiency in the presence of morphogens known to be either osteoinductive or chondrogenic in nature. Our primary findings (Additional file 1: Table S3) were that hydrodynamic loading, in the absence of exogenous supplementation, promotes expression of hypertrophic and osteogenic genes. Although not investigated in this study, we suspect this observation is due to integrin signaling activated by deformation of the MSC pericellular matrix in response to the hydrodynamic loads as indicated by other studies in the literature [30, 31]. The influence of hydrodynamic loading on osteogenic markers was sustained for low levels of exogenous supplementation irrespective of the cytokine provided. Lineage specific upregulation towards chondrogenic and osteogenic phenotypes was observed under high magnitude shear conditions when hydrodynamic loading was presented in concert with high levels of TGF-β3 and BMP-2 supplementation respectively. Generally, the observed impact of hydrodynamic loading on the desired phenotypes was greater in longer term cultures, and in cultures receiving higher concentrations of exogenous cytokines.

These findings are in agreement with prior mechanobiological studies in other osteochondral lineage cell sources. In studies based on osteoblastic cell lines, multiple studies have shown that hydrodynamic loading is osteopromotive [32–36], and often results in increases in type I collagen production and matrix mineralization. Hydrodynamic studies on MSCs from various donor species have also been previously shown to be osteoinductive [37–39]. Additionally, multiple studies on primary chondrocytes have shown that in addition to increases in type II collagen [40, 41]. Exposure to high shear environments can result in development of a fibrous layer rich in non-hyaline type I collagen at shear exposed surfaces [42], particularly when cultured with serum supplemented medium [43] versus serum free preparations. If we compare the extent of the impact of hydrodynamic loading on unsupplemented cultures in our study to that of other environmental induction schemes for MSCs, we find that the effect on gene expression is on the same order of magnitude as manipulations of scaffolding stiffness for osteoinduction [44] and both hydrostatic pressure [45] and dynamic unconfined compressive loading [46, 47] for chondrogenic induction. Unlike these prior studies, however, we found the impact of exogenous supplementation on gene expression to be considerably greater than the environmental stimulus applied. Our results converge again, however, when the mechanical stimuli were presented concurrently with TGF-β3 supplementation [45, 46]. As in our study utilizing hydrodynamic loading, dynamic compression and intermittent hydrostatic loading both resulted in additional increases in chondrogenic gene expression when presented in cultures supplemented with at least 10 ng/mL. The order of magnitude of the change, however, is considerably greater in our hydrodynamic study (>100 fold change) than either of the prior studies utilizing compressive (<10 fold change) and hydrostatic (<10 fold change) loading.

Conversely, other studies have shown compressive loading to have a negative impact on glycosaminoglycan accumulation within the construct at the protein level [48]. It is unclear, however, if this effect is due to decreased synthesis or loss of glycosaminoglycans to the culture medium. Interestingly, the same study [48], also showed that dynamic compressive loading resulted in increases in *COLXA1* in the absence of TGF-β3 supplementation, a result that mirrors our findings of both a hypertrophic influence of unsupplemented hydrodynamic loading and of the chondroprotective character of TGF-β3 supplementation. Our findings, herein, seem to indicate a comparable role of hydrodynamic loading to that of other environmental factors, particularly dynamic compression. Considering finite element analyses have shown interstitial fluid flow to be an effect of dynamic loading in biphasic materials such as those referenced herein, it is not surprising that these two loading conditions produce similar responses in MSC based tissue constructs.

Our finding that high magnitude hydrodynamic loading promotes osteogenic gene expression in unsupplemented cultures is instructive and suggests that MSCs cultures intended for chondral therapies not be subjected to high shear hydrodynamic loading conditions during processing and cultivation. While the modified parallel plate bioreactor system utilized better utilized for experimental investigation than a scalable manufacturing process, the bioprocessing principles derived from its use could easily be incorporated into a more scalable suspension bioreactor system based on three dimensional microcarriers. It is our recommendation that the nutrient utilization of chondrogenic cultures in such a system be carefully considered such that fluid loading not be applied in excess of magnitudes needed to meet the convective transport demands of the tissue in order to avoid potential induction of a hypertrophic phenotype. Conversely, our findings also suggest hydrodynamic loading of osteogenic cultures can potentially be a means of either reducing culture dependence on exogenous cytokines or promoting increased matrix deposition provided the magnitude of loading is increased such that impacts cell viability in a negative manner [49].

## Conclusions

The findings of this study bring forth a number of important considerations regarding hydrodynamic culture of MSC based constructs for tissue engineering applications. As evidenced by results from all cytokine supplementation groups, including serum-free expansion medium culture, it is clear that MSCs are tuned to their local mechanical loading environment, and that prolonged exposure to high magnitude fluid shear stresses induces a hypertrophic phenotype amongst the resident MSCs ultimately resulting in expression of osteogenic markers. For the purpose of chondrogenic cultures, therefore, our results suggest minimizing the fluid shear stress imposed on the developing construct without reducing the transport of nutrients to all regions of the tissue construct. Furthermore, this phenomenon presents an interesting paradigm for the production of osteochondral tissue constructs through differential loading of the construct, both chemically and hydrodynamically, by varying the microenvironment appropriately in spatially separated regions of the tissue construct. While the overall goal of the current study of a single medium source with differential loading to induce phenotypic changes in the MSC population was not achieved, there is evidence that loading will play a significant role in bioprocessing protocols of osteochondral constructs moving forward as technologies such as microfluidic hydrogels [50–53] provide the means to differentially apply chemical and environmental cues within an integrated construct of a single cell type to spatially engineer osteochondral tissues for intra-articular injury repair and preclinical models for pharmacological studies against osteoarthritis.

## Additional files

Additional file 1
**Table S1.** Experimental Condition Matrix. **Table S2.** RT-qPCR Primers. **Table S3.** Summary of Transcriptome Analysis after 2-Weeks of Culture. **Table S4.** Gene Regulation Ratios. (DOCX 21 kb) Additional file 2:
**Supplementary Methods.** (DOCX 17 kb) Additional file 3: Figure S1. Adherent cells were lifted from culture after one passaging and test for the presence of bovine MSC surface markers consisting of CD271, CD166, and CD44. Additionally, cells were tested for the absence of hematopoietic surface marker CD45. (TIF 2104 kb) Additional file 4: Figure S2. Following confirmation of MSC surface markers via flow cytometry, MSCs from the bone marrow of three calves were pooled, plated, cultured and assessed for tri-lineage differentiation potential. From left to right MSCs culture in inductive media (top) and growth medium (bottom row) were stained with Oil Red O, Toluidine Blue, and Alizarin Red to confirm evidence of adipogenesis, chondrogenesis, and osteogenesis respectively. (TIF 26421 kb)
