# Supplementary material for: Hydrodynamic loading in concomitance with exogenous cytokine stimulation modulates differentiation of bovine mesenchymal stem cells towards osteochondral lineages
Source: BMC Biotechnol. 2016 Feb 1;16:10. doi: 10.1186/s12896-016-0240-6 (PMC4736240; doi:10.1186/s12896-016-0240-6)
Supplement: Additional file 1 — Table S1. Experimental Condition Matrix. Table S2. RT-qPCR Primers. Table S3. Summary of Transcriptome Analysis after 2-Weeks of Culture. Table S4. Gene Regulation Ratios. (DOCX 21 kb) [file 12896_2016_240_MOESM1_ESM.docx]

| **Supplementation** | **Concentration** | **Wall Shear**  **Conditions** | **Durations** |
| --- | --- | --- | --- |
| TGF-β3 | 100 ng/mL | 0, 1, 10 dynes/cm^2^ | 0, 1, 2 Weeks |
|  | 10 ng/mL | 0, 1, 10 dynes/cm^2^ | 0, 1, 2 Weeks |
|  | 1 ng/mL | 0, 1, 10 dynes/cm^2^ | 0, 1, 2 Weeks |
| Unsupplemented | | 0, 1, 10 dynes/cm^2^ | 0, 1, 2 Weeks |
| BMP-2 | 1 ng/mL | 0, 1, 10 dynes/cm^2^ | 0, 1, 2 Weeks |
|  | 10 ng/mL | 0, 1, 10 dynes/cm^2^ | 0, 1, 2 Weeks |
|  | 100 ng/mL | 0, 1, 10 dynes/cm^2^ | 0, 1, 2 Weeks |

**Table S1: Experimental Condition Matrix**

**Table S3: Summary of Transcriptome Analysis after 2-Weeks of Culture**

| **Supplementation** | **Concentration [ng/mL]** | **Wall Shear  Condition [dynes/cm^2^]** | **sox9** | **aggrecan** | **col2a1** | **colXa1** | **col1a1** | **osteocalcin** | **runx2** |
| --- | --- | --- | --- | --- | --- | --- | --- | --- | --- |
|  |  |  |  |  |  |  |  |  |  |
| TGF-β3 | 100 | 10 | ↑ | ↑ | ↑ |  |  |  |  |
|  |  | 1 | ↑ | ↑ | ↑ |  |  |  |  |
|  |  | 0 | ↑ | ↑ | ↑ |  |  |  |  |
|  | 10 | 10 | ↑ | ↑ | ↑ |  |  |  |  |
|  |  | 1 | ↑ | ↑ | ↑ |  |  |  |  |
|  |  | 0 | ↑ | ↑ | ↑ |  |  |  |  |
|  | 1 | 10 |  |  | ↑ | ↑ |  |  |  |
|  |  | 1 |  |  |  | ↑ |  |  |  |
|  |  | 0 |  |  |  |  |  |  |  |
| Unsupplemented | | 0 |  |  |  |  |  |  |  |
|  |  | 1 |  |  |  |  |  |  |  |
|  |  | 10 |  |  |  | ↑ | ↑ |  | ↑ |
| BMP-2 | 1 | 0 |  |  |  |  |  |  |  |
|  |  | 1 |  |  |  |  |  |  |  |
|  |  | 10 | ↑ |  | ↑ | ↑ | ↑ |  | ↑ |
|  | 10 | 0 |  |  | ↑ | ↑ |  | ↑ |  |
|  |  | 1 |  |  | ↑ | ↑ |  | ↑ |  |
|  |  | 10 | ↑ |  | ↑ | ↑ | ↑ | ↑ | ↑ |
|  | 100 | 0 | ↑ | ↑ | ↑ | ↑ | ↑ | ↑ | ↑ |
|  |  | 1 | ↑ | ↑ | ↑ | ↑ | ↑ | ↑ | ↑ |
|  |  | 10 | ↑ | ↑ | ↑ | ↑ | ↑ | ↑ | ↑ |

Arrows indicate a statistically significant increase over Day 0 controls. Grey fillings indicate a statistically significant increase over supplementation and culture duration matched static cultures.

**Table S2: RT-qPCR Primers**

| **Function** | **Gene** | **Primer Nucleotide Sequence** | **Accession number from GenBank** |
| --- | --- | --- | --- |
| Housekeeping  Genes | ACTB | Forward 5’GAGCGGGAAATCGTCCGTGAC 3’  Reverse 5’ GTGTTGGCGTAGAGGTCCTTGC 3’ | NC007326 |
|  | GAPDH | Forward 5' CCTTCATTGACCTTCACTACATGGTCTA 3'  Reverse 5' TGGAAGATGGTGATGGCCTTTCCATTG 3' | U85042.1 |
| Chondrogenic  Markers | sox9 | Forward 5' CATGAAGATGACCGACGAG 3'  Reverse 5' CGTCTTCTCCGTGTCGGA 3' | AF278703.1 |
|  | aggrecan | Forward 5' CACTGTTACCGCCACTTCCC 3'  Reverse 5' GACATCGTTCCACTCGCCCT 3' | NM013227 |
|  | col2α1 | Forward 5’ ATCCATTGCAAACCCAAAGG 3’  Reverse 5’ CCAGTTCAGGTCTCTTAGAG 3’ | NM001113224.1 |
| Hypertrophic  Marker | colXα1 | Forward 5' CATGCTGCCACAAACAGC 3'  Reverse 5' TGGATGGTGGGCCTTTTA 3' | NC007307.5 |
| Osteogenic  Markers | runx2 | Forward 5' TTACAGACCCCAGGCAGGCACA 3'  Reverse 5' TCCATCAGCGTCAACACCATCA 3' |  |
|  | osteocalcin | Forward 5’ TGACAGACACACCATGAGAACCC 3’  Reverse 5’ AGCTCTAGACTGGGCCGTAGAAG 3’ | EF673278.1 |
|  | col1α1 | Forward 5' TGCTGGCCAACCATGCCTCT 3'  Reverse 5' CGACATCATTGGATCCTTGCA G 3' | AB008683 |

**Table S4: Gene Regulation Ratios**

|  | Ratio of Gene Expression Regulation | | | | | |
| --- | --- | --- | --- | --- | --- | --- |
|  | SOX9:RUNX2 | | | COL2A1:COL1A1 | | |
|  | 0 Week(s) | 1 Week(s) | 2 Week(s) | 0 Week(s) | 1 Week(s) | 2 Week(s) |
| BMP2-100-HighShear | 9.98E-01 | 4.89E-01 | 3.95E-01 | 1.00E+00 | 1.19E-02 | 2.01E-03 |
| BMP2-100-LowShear | 9.98E-01 | 4.79E-01 | 5.05E-01 | 1.00E+00 | 1.88E-02 | 1.54E-02 |
| BMP2-100-Static | 9.98E-01 | 5.06E-01 | 5.07E-01 | 1.00E+00 | 1.79E-02 | 4.49E-03 |
| BMP2-010-HighShear | 9.98E-01 | 5.17E-01 | 3.80E-01 | 1.00E+00 | 2.01E-02 | 8.11E-03 |
| BMP2-010-LowShear | 9.98E-01 | 6.73E-01 | 5.31E-01 | 1.00E+00 | 2.70E-02 | 1.65E-02 |
| BMP2-010-Static | 9.98E-01 | 2.50E-01 | 4.08E-01 | 1.00E+00 | 6.30E-02 | 8.29E-02 |
| BMP2-001-HighShear | 9.98E-01 | 1.74E+00 | 7.59E-01 | 1.00E+00 | 7.34E+00 | 3.26E+00 |
| BMP2-001-LowShear | 9.98E-01 | 1.77E+00 | 7.30E-01 | 1.00E+00 | 8.16E+00 | 2.85E+00 |
| BMP2-001-Static | 9.98E-01 | 2.03E+00 | 5.75E-01 | 1.00E+00 | 3.32E+00 | 1.00E+00 |
| SFCM-000-HighShear | 9.98E-01 | 5.37E-01 | 5.33E-01 | 1.00E+00 | 1.54E+00 | 1.00E+00 |
| SFCM-000-LowShear | 9.98E-01 | 8.13E-01 | 9.26E-01 | 1.00E+00 | 1.48E+00 | 1.27E+00 |
| SFCM-000-Static | 9.98E-01 | 5.15E-01 | 6.34E-01 | 1.00E+00 | 2.07E+00 | 1.43E+00 |
| TGFB3-001-Static | 9.98E-01 | 8.50E-01 | 4.22E-01 | 1.00E+00 | 8.56E-01 | 7.58E-01 |
| TGFB3-001-LowShear | 9.98E-01 | 1.95E+00 | 5.81E-01 | 1.00E+00 | 5.42E+00 | 3.37E+00 |
| TGFB3-001-HighShear | 9.98E-01 | 1.59E+00 | 7.26E-01 | 1.00E+00 | 1.13E+01 | 5.03E+00 |
| TGFB3-010-Static | 9.98E-01 | 6.52E+00 | 6.62E+00 | 1.00E+00 | 3.48E+01 | 2.26E+01 |
| TGFB3-010-LowShear | 9.98E-01 | 2.59E+01 | 1.61E+01 | 1.00E+00 | 1.06E+01 | 6.84E+00 |
| TGFB3-010-HighShear | 9.98E-01 | 9.44E+01 | 7.78E+01 | 1.00E+00 | 7.27E+01 | 3.61E+01 |
| TGFB3-100-Static | 9.98E-01 | 1.07E+01 | 1.32E+01 | 1.00E+00 | 3.38E+01 | 2.14E+01 |
| TGFB3-100-LowShear | 9.98E-01 | 4.67E+01 | 2.69E+01 | 1.00E+00 | 1.75E+02 | 1.90E+02 |
| TGFB3-100-HighShear | 9.98E-01 | 2.22E+02 | 1.14E+02 | 1.00E+00 | 3.51E+02 | 3.50E+02 |
