# Supplementary material for: Hydrodynamic loading in concomitance with exogenous cytokine stimulation modulates differentiation of bovine mesenchymal stem cells towards osteochondral lineages
Source: BMC Biotechnol. 2016 Feb 1;16:10. doi: 10.1186/s12896-016-0240-6 (PMC4736240; doi:10.1186/s12896-016-0240-6)
Supplement: Additional file 2: — Supplementary Methods. (DOCX 17 kb) [file 12896_2016_240_MOESM2_ESM.docx]

# Supplementary Methods

## MSC Isolation

Bovine bone marrow aspirates were harvested from within the subchondral trabecular bone of the femoral condyles of the cadaveric limbs from 2–4 week old calves within 48 hours of slaughter (Research 87, Marlborough, MA). Isolated marrow was mixed with expansion medium (high glucose Dulbecco’s Modified Eagle Medium [DMEM] supplemented with 10% certified fetal bovine serum [FBS] and 1× penicillin-streptomycin-fungizone [PSF]) supplemented with 300 U/ml heparin), vortexed to remove any undesirable fat and bone fragments from the marrow, passed through a 60µm cell strainer, and centrifuged to collect cell pellets. Cells were resuspended in the expansion medium and plated onto T-75 flasks (Corning, Inc., Corning, NY). After an initial period of 24 hours, non-adherent cells were removed from the flasks, whereas adherent cells were cultured in expansion medium for an additional 7–10 days until cultures reached confluence. Subsequent subculturing was carried out to Passage 3 at a splitting ratio of 1:3. Following Passage 3, MSCs that were suspended in a cryoprotective medium (70% DMEM, 20% FBS, 10% dimethylsulfoxide, 1X PSF) at a concentration of 1 million cells/mL and stored in liquid nitrogen in 1mL aliquots.

## MSC Characterization

Following Passage 1, a portion of MSCs were fixed, treated with a nonspecific blocking agent for 30 minutes, and split into six tubes. Four of the six populations were then incubated with one of the following fluorescently tagged antibodies: fluorescein isothiocyanate [FITC]-conjugated mouse anti-human antibodies against each of CD166, CD271, and CD45, or R-phycoerythrin [RPE]-conjugated mouse anti-bovine CD44 antibodies for 1 hour. The remaining two populations were incubated with FITC and RPE conjugated antibodies against mouse IgG as the negative isotype controls. Flow cytometry was performed in a FACScan (BD Biosciences, San Jose, CA). Forward scatter and side scatter parameters were used to evaluate the size and granularity of cells, respectively. Flow cytometric analysis showed a consistent expression of MSC surface markers (CD166, CD271, CD44) while being negative for CD45, a key hematopoietic stem cell marker (**Supplementary Figure 1**).

Following confirmation of a consistent set of multipotent cell surface markers, MSCs were plated in a 12 well plate at a seeding density of 100,000 cell/well in one of four culture media preparations: expansion medium (EM), osteogenic medium (OM), adipogenic medium (AM), and chondrogenic medium (CM). Osteogenic medium consisted of high glucose DMEM supplemented with 10% FBS, 1X PSF, 100 nM dexamethasone, 10 mM sodium β-glycerophosphate, 0.05 mM ascorbic acid. Adipogenic medium consisted of high glucose DMEM supplemented with 10% FBS, 1X PSF, 1 μM dexamethasone, 0.5 mM indomethacin, 10 μg/ml insulin, 100 mM 3-isobutyl-1-methylxantine. Chondrogenic medium consisted of high glucose DMEM supplemented with 1× PSF, 0.1 µM dexamethasone, 50 µg/mL ascorbate 2-phosphate, 40 µg/mL l-proline, 100 µg/mL sodium pyruvate, 1X insulin–transferrin–selenium [ITS], and 10 ng/mL TGF-β3. Following 21 days of culture, the monolayers were fixed and assessed for successful induction. Osteogenesis was determined by fixing monolayers in isopropanol and staining with Alizarin Red for mineralized matrix. Adipogenesis was assessed by fixing with paraformaldehyde and staining with freshly prepared Oil Red O to visualize lipid droplets. Chondrogenesis was determined by fixing monolayers with 10% formalin and staining with Toluidine Blue for an abundance of proteoglycans (**Supplementary Figure 2**).
