# Supplementary figures and images for: Hydrodynamic loading in concomitance with exogenous cytokine stimulation modulates differentiation of bovine mesenchymal stem cells towards osteochondral lineages
Source: BMC Biotechnol. 2016 Feb 1;16:10. doi: 10.1186/s12896-016-0240-6 (PMC4736240; doi:10.1186/s12896-016-0240-6)

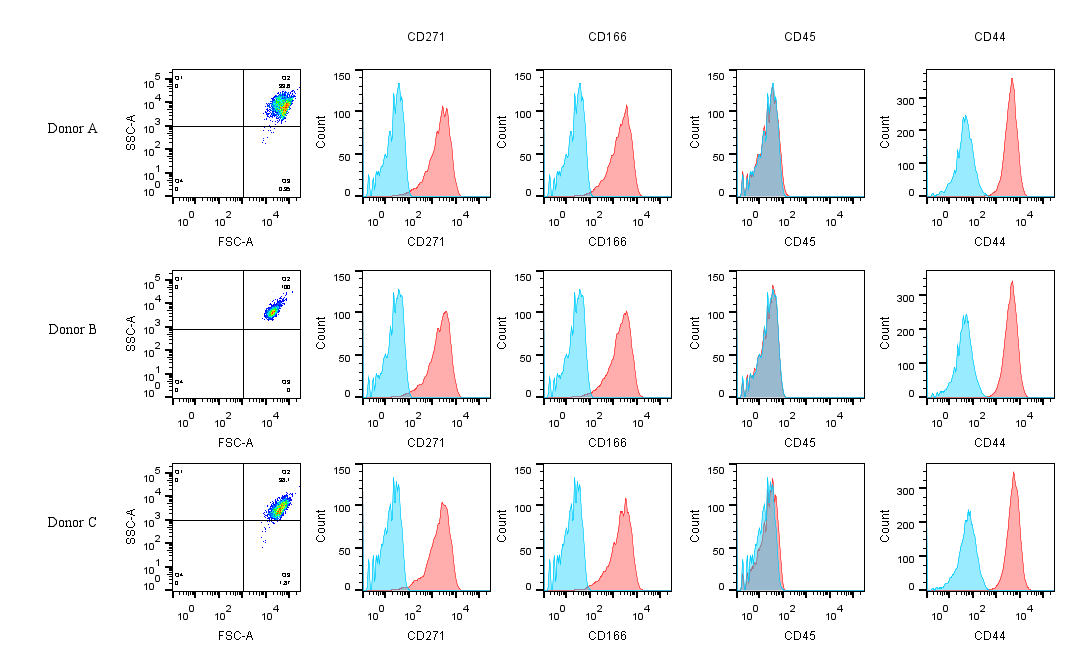

Supplement: Additional file 3: Figure S1. — Adherent cells were lifted from culture after one passaging and test for the presence of bovine MSC surface markers consisting of CD271, CD166, and CD44. Additionally, cells were tested for the absence of hematopoietic surface marker CD45. (TIF 2104 kb) [file 12896_2016_240_MOESM3_ESM.tif]

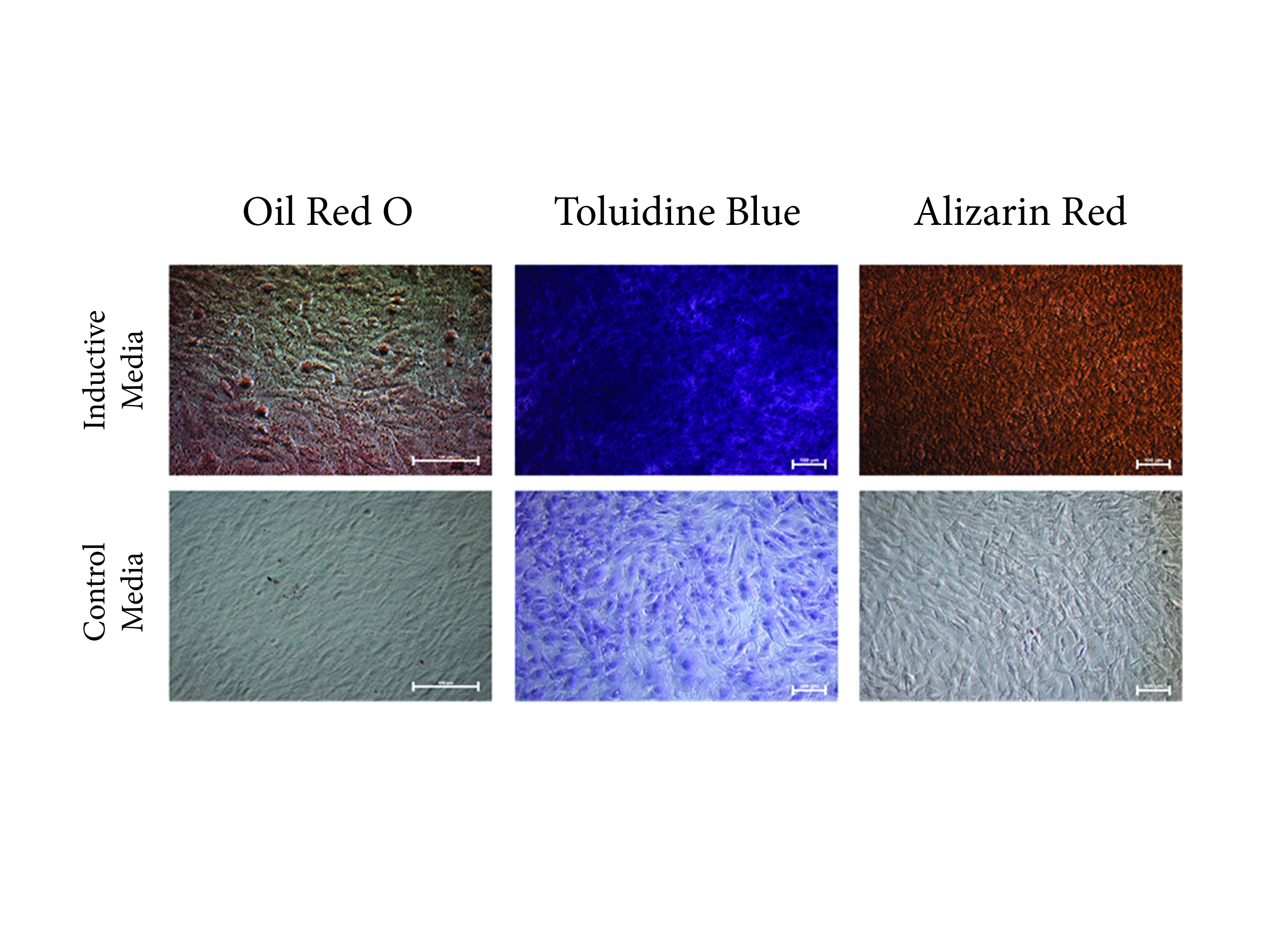

Supplement: Additional file 4: Figure S2. — Following confirmation of MSC surface markers via flow cytometry, MSCs from the bone marrow of three calves were pooled, plated, cultured and assessed for tri-lineage differentiation potential. From left to right MSCs culture in inductive media (top) and growth medium (bottom row) were stained with Oil Red O, Toluidine Blue, and Alizarin Red to confirm evidence of adipogenesis, chondrogenesis, and osteogenesis respectively. (TIF 26421 kb) [file 12896_2016_240_MOESM4_ESM.tif]
